# GABA content and an antioxidant profile positively correlated with the anticonvulsive activity of Microcos paniculata in acute seizure mice

**DOI:** 10.1016/j.heliyon.2023.e18295

**Published:** 2023-07-14

**Authors:** S.M. kamruzzaman, Latifa Bulbul, Md Zahir Alam, Md Mostafizur Rahman

**Affiliations:** a Department of Pharmacy, Noakhali Science and Technology University, Noakhali, Bangladesh; b Department of Horticulture, Sher-e-Bangla Agricultural University, Dhaka, Bangladesh; c Department of Chemistry, National University, Dhaka, Bangladesh

**Keywords:** Epilepsy, GABA, Antioxidant enzymes, Lipid peroxidation, Plant extracts

## Abstract

This study evaluated the effects of different parts of *M. paniculata* (MP) extracts on convulsions and antioxidant activities in mice. Six polyphenolic compounds were identified, where epicatechin and quercetin have been identified in the highest amounts (23.01 and 32.23 mg/100 g of dry MP extract, respectively) in MP leaf and stem extracts, using Ultra Performance Liquid Chromatography. 7-day oral administration of MP at doses of 100, 200, and 400 mg/kg body weight (BW) significantly reduced convulsions and reduced mortality rates compared with seizure inducer groups. Antioxidant potentials were measured by superoxide dismutase (SOD), catalase (CAT), thiobarbituric acid reactive substances (TBARS), and reduced glutathione (GSH) content in whole-brain homogenates. Gamma-aminobutyric acid (GABA) levels significantly increased in leaves and stem-treated groups, suggesting that MP leaves and stems have potent antioxidant properties that can attenuate convulsions by modulating the GABAergic system and antioxidant activities.

## 1 Introduction

Epilepsy is a neurological disorder characterized by recurrent, unprovoked seizures due to abnormal neuronal firing in the brain. Seizures manifest as convulsions, loss of consciousness, and brief periods of gazing or confusion [1,2]. It is the second most common neurological disorder [3], and approximately 80% of people with epilepsy live in developing countries with little or minimal access to adequate medical treatment or therapy [4]. Numerous classes of anticonvulsive drugs are presently available, but they cannot control seizures effectively [5]. Besides, the dose-related neurotoxicity and other side effects connected with anticonvulsive drugs limit their clinical utilization [5,6]. However, the problem of adverse effects has also not been dodged completely, and approximately 30% of the patients continue to have seizures with current anticonvulsive drug therapy [7]. Hence, research should continue to develop newer, more effective, and more secure neuroprotective agents for the treatment of epilepsy. Medicinal plants used in traditional medicine for the therapy of epilepsy have scientifically appeared to possess promising anticonvulsant activities with lesser side effects in animal models for screening for anticonvulsant activity [8].

*M. paniculata* L. (Family-Tiliaceae) is a shrub or small tree, locally known in Bangladesh as ‘Kathgua’ or ‘Fattashi’ [9], and cultivated in Bangladesh, Sri Lanka, India, Indonesia, Myanmar, Malaysia, China, the Andaman Islands, Vietnam, Cambodia, and Thailand. It has been used ethnomedicinally for a wide range of activities, including, antipyretic [10], dyspepsia [11], analgesic, anti-inflammatory [12], neuropharmacological, free radical scavenger [13], α-glucosidase inhibition, nicotinic receptor antagonistic activities, as well as treating heart diseases. The antioxidant activities of phenolic components from various plants of Microcos species have been studied [14]. Previous studies showed that MP extracts displayed significant free-radical-scavenging activity, which is similar to that of the standard antioxidant ascorbic acid and, therefore, may be a promising natural antioxidant [15]. Recently, the anticonvulsive activity of different parts of the MP extract in mice was studied in our lab, and it possesses excellent anticonvulsant activity. Moreover, an acute toxicity study of MP extract was also conducted, and mortality signs of any toxicity or behavioral changes were not observed up to a dose as high as 4000 mg/kg [11].

Oxidative injury may play a role in the initiation and progression of epilepsy; therefore, therapies aimed at reducing oxidative stress may remediate tissue damage and favorably alter the clinical course [16]. Membrane lipid peroxidation caused by excessive free radical generation or a decrease in the activities of antioxidant defense systems has been suggested to be critically involved in seizure control [17]. Several studies in animals and humans have demonstrated that certain anticonvulsive drugs exhibit antioxidant effects by modulating the activity of various enzymes associated with this type of stress [18]. Considering the data mentioned above, we aimed to compile the existence of a possible correlation between seizure-related alterations in GABA content and brain oxidative status. Hence, the present study was undertaken to evaluate the relevance of the antioxidant properties to the anticonvulsant activities of MP plant extracts.

## 2 Materials and methods

### 2.1 Plant material and extraction

MP was collected from Daudkandi, Comilla, Bangladesh. A voucher specimen for these plants has been collected from the Bangladesh National Herbarium, Dhaka, Bangladesh (Microcos paniculata, Accession Number 39542). The collected plant parts were extracted by maceration with 1200 ml of 90% methanol (Merck, Darmstadt, Germany) and evaporated using a rotary evaporator. 14.34 g (Yield 4.78%) of leaves, 9.06 g (Yield 3.02%) of flowers, 11.05 g (Yield 3.68%) of roots, and 12.21 g (Yield 4.07%) of stems were obtained. These crude extracts were stored in a well-closed glass container at 5 °C in a refrigerator for the desired studies.

### 2.2 Animals

Swiss-albino mice of either sex, aged 4–5 weeks (25 ± 2g) obtained from the laboratory mice-breeding centre in the Department of Pharmacy at Jahangirnagar University, were used for the studies. All animal experiments were performed in accordance with the Animal Ethical Committee of Noakhali Science and Technology University, Noakhali (Reference: NSTU/SCI/EC/2023/125(A). The extracts of MP were dissolved in dimethyl sulfoxide (DMSO) and orally administered to the test animals for 6 days and on days 7, 1 h before the experiments at doses of 100, 200, and 400 mg/kg BW in both the chemical-induced convulsion and electric shock-induced convulsion models. Mice were randomly divided into 15 groups (n = 5/group), where groups were considered as follows: GI: control group (distilled water 10 ml/kg BW by oral route); GII: seizure inducer group (distilled water + electrical shock, isoniazid, or pilocarpine); and GIII: standard treated group (phenytoin or diazepam). Plant parts are divided as follows (Table 1).

**Table 1:** Groups of different parts of MP with respective doses amount.

Dose (mg/kg BW)	Leaves	Roots	Flowers	Stems
100	GIV	GVII	GX	GXIII
200	GV	GVIII	GXI	GXIV
400	GVI	GIX	GXII	GXV

### 2.3 Ultra-performance liquid chromatography (UPLC) of polyphenolic compound

#### 2.3.1 Preparation of standards and samples

Each stock standard phenolic compound solution (100 μg/ml) was prepared in methanol by dissolving 0.005 g of the analyte into a 50 ml volumetric flask. For preparing a mixed standard solution, the stock standard phenolic solutions were diluted in methanol to give a concentration of 5 μg/ml for each polyphenol except (+) CH, CA, RH (4 μg/ml) and QU (3 μg/ml). All standard solutions were stored in the dark at 5 °C to reduce reactions. All extract solution was prepared in methanol at a concentration of 5 mg/ml by using a vortex mixer for 30 min and stored at a low temperature (5 °C). The sample solution with phenolic standards was spiked for additional identification of individual polyphenols. All solutions (mixed standards, samples, and spiked solutions) were filtered through a 0.20 μm nylon syringe filter (Sartorius, Germany) before UPLC analyses and then degassed for 15 min in an ultrasonic bath (Hwashin, Korea).

#### 2.3.2 Conditions of chromatogram

The phenolic composition of MP was determined by a previously described UPLC protocol (Chuanphongpanich and Phanichphant, 2006) with some modifications [19]. Acetonitrile (solvent A), acetic acid solution at pH 3.0 (solvent B) and methanol (solvent C) were used as mobile phases. The system was run with the following gradient elution program: 0 min, 5%A/95%B; 10 min, 10%A/80%B/10%C; 20 min, 20%A/60%B/20%C, and 30 min, 100%A. A 5 min post run was practiced at initial conditions for equilibration of the column. 20 μl of MP was injected, and the flow rate was kept constant throughout the analysis at 1 ml/min. The wavelength program was optimized to monitor phenolic compounds at their respective maximum absorbance wavelengths (*λ*_max_) as follows: 280 nm was held for 19 min, changed to 320 nm and held for 7 min and finally changed to 380 nm for the UV detection and held for the rest of the analysis. The DAD was set in an acquisition range from 200 to 700 nm. The detection and quantification of gallic acid (GA), catechin (CH), vanillic acid (VA), caffeic acid (CA), and epicatechin (ECH) were done at 280 nm; ferulic acid (FA), vitexin (VN), apigenin (AP), P-coumaric acid (PCA), and rutin hydrate (RH) at 320 nm; and myricetin (MC), quercetin (QU), and kaempferol (KF) at 380 nm.

#### 2.3.3 Peak analysis

Ultra-performance liquid chromatography allows for highly sensitive and selective high-throughput quantification of plant antioxidant compounds [20]. The compounds were identified by comparison of the retention time and the absorbance spectrum profile of standards with each identified compound using Dionix Chromeleon software. They were also identified by running the samples after adding pure standards. From the calibration curves, each determined compound was quantified. The calibration curves of the standards were made by five sets of dilutions of the stock standards using methanol to yield 1.0–5.0 μg/ml for GA, VA, ECH, FA, VN, AP, PCA, MC, and KF; 0.5–4.0 μg/ml for CH, CA, and RH, and 0.25–3.0 μg/ml for QU. The calibration curves were constructed by plotting the peak area from the chromatogram versus the concentration of the standard with an R^2^ greater than 0.995. Data were represented as means ± SDs of triplicate independent analyses.

### 2.4 Anticonvulsant screening method

#### 2.4.1 Maximum electroshock (MES) induced convulsions model

The electroshock was applied separately to each mouse via a pair of corneal electrodes. The stimulus duration was 0.2 s, and the current frequency was 50 mA. All extracts were administered for seven days, and on an experimental day, the test started 60 min after administration of distilled water and extracts and 30 min after the standard drug (phenytoin 25 mg/kg, injected intraperitoneally (i.p.)). Electroshock was applied to trigger generalized tonic-clonic seizures in mice [21,22] and was observed for the occurrence of tonic hind limb extension (THLE) and mortality for a duration of 15 min [23]. The percentages of deaths occurring in the GII group were taken as 100%.

#### 2.4.2 Chemical-induced convulsion

##### 2.4.2.1 Isoniazid (INH) induced convulsions model

After 1 h of distilled water (GII group) and extract administration and 30 min of diazepam (5 mg/kg i. p.) administration, INH (300 mg/kg, i. p.) was injected into the mice to induce convulsions. Then each mouse was placed in an isolated plexiglas chamber and observed for the following 30 min for the onset and duration of convulsions and the mortality rate. Seizures were defined according to the Racine scale with minor modifications [[24], [25], [26]]. Seizure severity was measured up to the point where the maximal effect was reached using the following stages: stage 1: fictive scratching; stage 2: tremors, head nodding; stage 3: forelimb clonus; stage 4: continuous rearing, falling; stage 5: tonic-clonic seizures with hind limb extension, stupor, or jumping. The percentages of deaths occurring in the GII group were taken as 100% [27].

##### 2.4.2.2 Pilocarpine (PLO)- induced convulsions model

Pilocarpine-induced convulsion is a commonly used seizure model in rodents. In the current study, PLO-induced convulsions were evaluated according to previously described methods [28,29] with slight modifications. After 1 h of distilled water and extract administration and 30 min after diazepam administration into mice, hyoscine butyl bromide (1 mg/kg i. p.) was administered to reduce peripheral autonomic effects, induced by PLO in the animals. After injecting hyoscine butyl bromide, PLO (240 mg/kg, i. p.) was administered into mice of GII at 15 min and GIII to XV at 30 min, respectively. The animals were then placed in an isolated plexiglas testing chamber and recorded the onset time (the first episode of forelimb clonus) and duration of seizure, as well as the mortality rate for the following 30 min according to the scale described in the INH-induced convulsions model. The status epilepticus has been characterized as a convulsion at stages 4 and 5, as described previously [24,30].

### 2.5 Biochemical estimation

After the completion of behavioral experiments, mice were sacrificed, and the whole intact brain was carefully removed and incubated in an ice-chilled compartment for cleaning. The cerebellum was detached immediately, while the rest of the brain tissue was homogenized in a phosphate buffer (pH 7.6) and centrifuged at 20,000×*g* at 4 °C for 2 h.

#### 2.5.1 Catalase assay (CAT)

CAT activities were analyzed with a reaction solution containing: 2.5 ml of 50 mmol phosphate buffers (pH 5.0), 0.4 ml of 5.9 mmol hydrogen peroxide (H_2_O_2),_ and 0.1 ml of tissue homogenate. Changes in the absorbance of the reaction solution at 240 nm were recorded after 1 min. One unit of CAT activity was expressed as an observable change of 0.01 as a unit/mg protein [31].

#### 2.5.2 Superoxide dismutase assay (SOD)

SOD activity was evaluated by the method of Kakkar et al. [32]. The reaction mixture of this method consisted of 0.1 ml of phenazine methosulphate (186 μmol), 1.2 ml of sodium pyrophosphate buffer (0.052 mmol; pH 7.0), 0.3 ml of the supernatant after centrifugation (1500×*g* for 10 min followed by 10,000×*g* for 15 min) of the homogenate, which was incorporated into the reaction mixture. The enzyme reaction was started by adding 0.2 ml of NADH (780 μmol) and ended after 1 min by adding 1 ml of glacial acetic acid. The amount of chromogen formed was measured by recording the colour intensity at 560 nm. Results are shown in unit/mg protein.

#### 2.5.3 Reduced glutathione assay (GSH)

GSH was evaluated by the method described by Mitchell et al. [33]. A 1.0 ml sample of 10% brain homogenate was precipitated with 1.0 ml of 4% sulfosalicylic acid (C_7_H_6_O_6_S). The samples were kept at 4 °C for 1 h and then centrifuged at 1200×*g* at 4 °C for 20 min. The total volume of the 3.0 ml assay mixture contained a 0.1 ml filtered aliquot, 2.7 ml phosphate buffer (0.1 M, pH 7.4) and 0.2 ml of 100 mM DTNB (5, 5-dithiobis-2-nitrobenzoic acid). When the yellow colour of the mixture progressed, the absorption was measured at 412 nm on the spectrophotometer, and the levels of GSH were expressed as nmol GSH/mg tissue.

#### 2.5.4 Estimation of lipid peroxidation in the brain (TBARS)

The lipid peroxidation assay is known as thiobarbituric acid reactive substances and was conducted following the modified method of Ohkawa et al. [34]. The reaction mixture comprised a volume of 1.0 ml, which accommodated 0.58 ml phosphate buffer (0.1 mol; pH 7.4), 0.2 ml homogenated brain sample, 0.2 ml ascorbic acid (100 mmol) and 0.02 ml ferric chloride (100 mmol). The reaction mixture was incubated for 1 h in an agitating water bath at 37 °C. The reaction was stopped by the addition of 1.0 ml of 10% trichloroacetic acid. After that, 1.0 ml 0.67% thiobarbituric acid was added and all the tubes were placed in a boiling water bath for 20 min, and then transferred into a crushed ice-cold bath before centrifuging at 2500×*g* for 10 min. The amount of TBARS reduced in each of the samples was estimated by the absorbance of the supernatant at 535 nm using a UV spectrophotometer against a reagent blank. The results were expressed as nmol TBARS/mg tissue at 37 °C using a molar extinction coefficient of 1.56 × 105 M^−1^ cm^−1^.

#### 2.5.5 Estimation of brain gamma aminobutyric acid (GABA) content

The brain's GABA content was estimated according to the method of Holdiness et al. [35]. A sample (0.1 ml) of brain homogenate was placed in 0.2 ml of 0.14 M ninhydrin solution in 0.5 M carbonate-bicarbonate buffer (pH 9.95), kept in a water bath at 60 °C for 30 min, then cooled and treated with 5 ml of copper tartrate reagent, which contained 0.16% Na_2_CO_3_, 0.03% copper sulphate and 0.0329% tartaric acid (C_4_H_6_O_6_). After 10 min, fluorescence at 377/455 nm in a spectrofluorometer was recorded.

### 2.6 Statistical analysis

For phytochemical analysis, data were represented as means ± SDs of triplicate independent analyses. All other values were expressed as mean ± SEM. The behavioral and biochemical parameter data were analyzed using ANOVA followed by Dunnett's test. *P values < 0.05, **P values < 0.01, and ***P values < 0.001 were considered significant.

## 3 Results

### 3.1 Determination of total phenolic contents (TPC) and total flavonoid contents (TFC)

The MP was evaluated for their phenolic contents by the Folin-Chiocalteu assay.

The methanol leaf extract showed the highest phenolic content (220 ± 2.35 mg GAE/g of dried extract). The roots, flowers, and stems extracts evaluated for phenolic contents showed maximum values of 37.6 ± 2.12, 130 ± 2.33, 162 ± 2.65 mg GAE/g of dried extract, respectively. The root extract of MP showed the lowest values of phenolic contents, with 13 ± 1.53 mg GAE/g of dried extract. The flavonoid contents of MP extracts were expressed as quercetin equivalents in mg QE/g of dry weight. The flavonoids contents for MP extracts of leaves, roots, flowers, and stems were found to be 220 ± 2.35, 37.6 ± 2.12, 130 ± 2.33 and 162 ± 2.65 mg QE/g, respectively (Table 2).

**Table 2:** Total phenolic and flavonoid content determination of methanolic extract of MP.

Treatment	Conc. mg/ml	Total phenol content of methanol extract of MP mg Gallic acid equivalent (GAE) per mg sample, y = 0.007x + 0.036, R^2^ = 0.997	Total flavonoid content of methanol extract of MP mg quercetin equivalent per μg y = 0.006x + 0.056, R^2^ = 0.992
Leaves	0.5 mg/ml	74 ± 1.528	45 ± 0.577
	0.75 mg/ml	103 ± 1.326	126 ± 1.856
	1 mg/ml	220 ± 2.35	217 ± 3.383
Roots	0.5 mg/ml	13 ± 1.528	11 ± 2.082
	0.75 mg/ml	23.6 ± 0.186	21.8 ± 2.581
	1 mg/ml	37.6 ± 2.122	45.2 ± 3.176
Flowers	0.5 mg/ml	26 ± 1.155	23 ± 1.732
	0.75 mg/ml	73.7 ± 1.764	30 ± 0.765
	1 mg/ml	130 ± 2.333	56.7 ± 2.943
Steam	0.5 mg/ml	29.00 ± 1.00	28 ± 1.155
	0.75 mg/ml	66.3 ± 0.333	50.3 ± 1.453
	1 mg/ml	162 ± 2.646	120 ± 4.726
Gallic acid		260 ± 2.35	–
Quercetin		–	241 ± 3.38

Data represents mean ± standard deviation (n = 3) of triplicate analysis.

### 3.2 Ultra-performance liquid chromatography chromatogram of MP extract

**Fig. 1:**
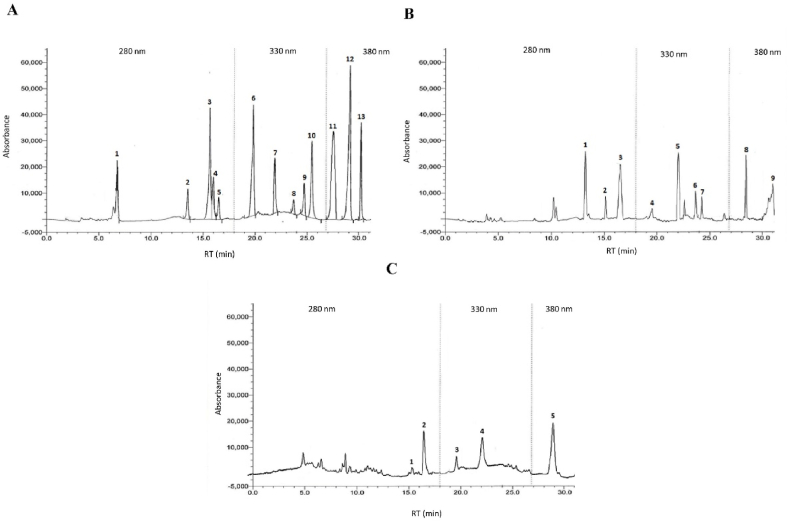
Ultra-performance liquid chromatography chromatogram of a standard mixture, leaf extract, and stem extract of polyphenolic compounds. **A)** Ultra-performance liquid chromatography chromatogram of a standard of polyphenolic compounds. Peaks: 1, gallic acid; 2, (+)-catechin; 3, vanillic acid; 4, caffeic acid; 5, (−)-epicatechin; 6, ferulic acid; 7, vitexin; 8, apegenin; 9, P-coumaric acid; 10, rutin hydrate; 11, myricetin; 12, quercetin; 13, kaempferol. **B)** Ultra-performance liquid chromatography chromatogram of MP leaf extracts of polyphenolic compounds. Peaks: 1, (+)-catechin; 2, vanillic acid; 3, (−)-epicatechin; 4, ferulic acid; 5, vitexin; 6, apigenin; 7, P-coumaric acid; 8, quercetin; 9, kaempferol; **C)** Ultra-performance liquid chromatography chromatogram of an MP stem extract of polyphenolic compounds. Peaks: 1, vanillic acid; 2, (−)-epicatechin; 3, ferulic acid; 4, vitexin; 5, quercetin.

The qualitative high-performance liquid chromatography (HPLC) analysis of crude methanol extracts of MP leaves and stems was subjected to evaluation, as they showed good anticonvulsant activity and contained the highest phenolic and flavonoid contents. *The chromatographic separations of the polyphenols of the standard mixture and MP leaf and MP stem extract were shown in* Fig. 1A and B, *and C, respectively, and the content of each phenolic compound was presented as the mean of three determinations (*Table 3). The experimental results indicate the presence of an especially high concentration of VN, KF, QU, AP, ECH, and CH of 42.21, 40.51, 39.05, 35.13, 30.24, and 23.43 mg per 100 g of dry MP weight, respectively, in the leaf extract. VA, PCA, and FA are detected in the extract but in lower amounts (14.20, 16.03, and 19.48 mg per 100 g of dry MP weight, respectively). On the other hand, QU and ECH were present at higher concentrations of 32.23 and 23.01, respectively, whereas the lowest amounts of VA, FA, and VN were found in stem extract at concentrations of 9.01, 16.02, and 13.21, respectively.

**Table 3:** Polyphenolic compounds in the MP leaves and stems extracts.

Polyphenolic compounds	Wavelength (λ) nm	RT (min)	Avg. area Mix standard	MP Leaves Extract			MP Stems Extract		
				Avg. area MP	*Content (mg/100 g of dry extract)*	*% RSD*	Avg. area MP	*Content (mg/100 g of dry extract)*	*% RSD*
GA	280	6	3864	–	–		–	–	–
CH	‘’	13	5096	10556	23.43	2.24	–	–	–
VA	‘’	15	17640	3780	14.20	1.36	2940	9.01	0.85
CA	‘’	16	4032	–	–	–	–	–	–
ECH	‘’	16.2	19504.8	8618.4	30.24	2.89	8164.8	23.01	2.16
FA	320	19.2	13516.8	2457.6	19.48	1.86	4915.2	16.02	1.51
VN	‘’	22	14784	16896	42.21	4.03	7744	13.21	0.96
AP	‘’	23.3	3728	8947.2	35.13	3.35	–	–	–
PC	‘’	24.3	10108.8	7776	16.03	1.53	–	–	–
RH	‘’	25	22400	–	–		–	–	–
MC	380	27	32832	–	–		–	–	–
QU	‘’	28.3	59147	23658.8	39.05	3.73	21508	32.23	3.13
KF	‘’	30	43320	13680	40.51	3.87	–	–	0.85

RT, retention time; Avg., Average; n = 3; MP, *M. paniculata*; RSD, Relative standard deviation; GA, gallic acid; CH, (C)-catechin hydrate; VA, vanillic acid; CA, caffeic acid; ECH, (−)-epicatechin; FA, ferulic acid; VN, vitexin; AP, apigenin; PCA, p-coumaric acid; RH, rutin hydrate; MC, myricetin; QU, quercetin; KF, kaempferol.

### 3.3 Anticonvulsant activity

#### 3.3.1 Effects of pretreated on MES, INH, and PLO-induced convulsions in mice

**Fig. 2:**
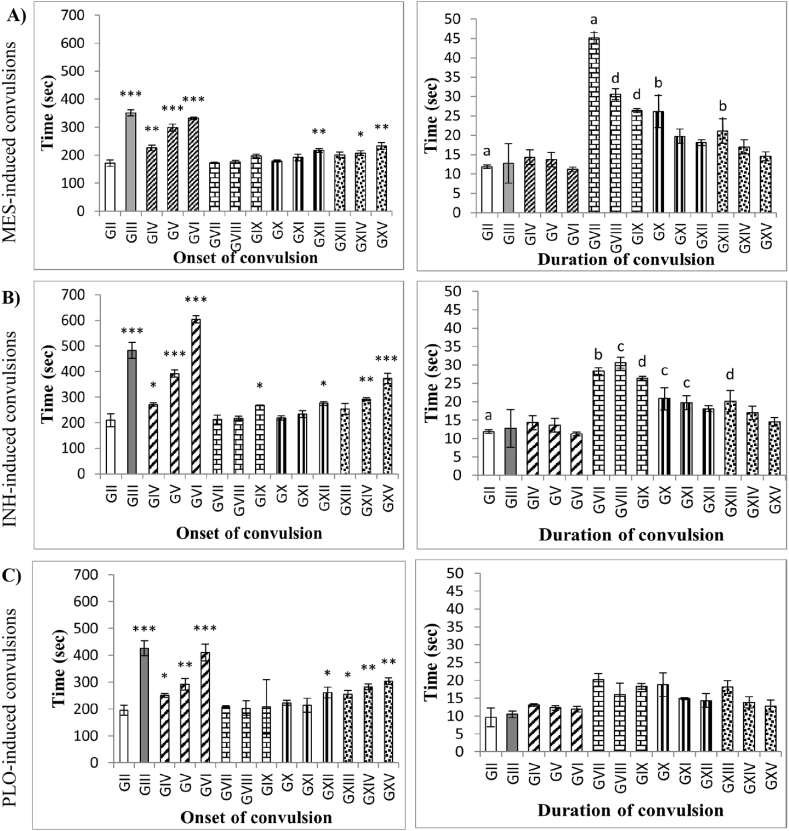
The effect of oral administration of different doses of extract on MES, INH, and PLO-induced convulsions in mice. Onset and duration of **A)** MES **B)** INH, and **C)** PLO-induced convulsion following MP administration. Values are expressed as the mean ± SEM (n = 5). Superscript stars represent the statistical significance determined by ANOVA followed by Dunnett's test. *p < 0.05, **p < 0.01, and ***p < 0.001 indicate significant differences from the seizure inducer groups. Mortality rate: a = 100%, b ≥ 70%, c ≥ 40%, d ≥ 10%; no alphabets = no death.

*In the electric shock and chemical-induced convulsion models, the initiation time of convulsion was found to be the highest with the leaf extract administration.* The anticonvulsant activity of leaf, stem, flower, and root extracts of MP was evaluated using three seizure-induced convulsion models. The MP extract 100, 200, and 400 mg/kg doses showed dose-dependent increases in the onset of convulsion compared to the distilled water + seizure inducer groups (Fig. 2 A, B, and C). In the MES-induced seizure model, the onset of convulsion increased significantly by (32.07–93.69%) in leaf, 26.4% in flowers, and (17.23–35.98%) in stem compared to the distilled water + MES group. The onset time increased to 105% (p < 0.001) with standard compared to the distilled water + MES group (Fig. 2A). In the INH-induced convulsion model, the onset of convulsion increased significantly by (28.84–187.4%) in leaf, 27.41% in root, 31.15% in flowers, and (38.88–77.53%) in stem relative to the distilled water + INH group. The onset time increased to 129.3% (p < 0.001) with standard compared to the distil water + INH group (Fig. 2B). In the PLO-induced SE model, the onset of convulsion increased significantly by (28.79–110.8%) in leaf, 34.5% in flowers, and (30.9–56.04%) in stem relative to the distil water + PLO group. The onset time increased to 119% (p < 0.001) with standard compared to the distilled water + PLO group (Fig. 2B).

In the three-seizure model, at a dose of 400 mg/kg, the highest onset of convulsion time was observed in leaf extracts (p < 0.001) among other parts of plant extracts. In all three-seizure models, the onset of convulsion increased in the order of leaves > stem > flower > root. However, extracts were incapable of reducing convulsion duration compared to the seizure-inducer groups. The MP leaves and stem prevent mortality in all three seizure models, with the exception of the stem extract at a dose of 100 mg/kg BW, in which 70% and 10% of mice died following MES- and INH-induced convulsions, respectively. Whereas flower and root extracts were unable to protect mice from death (10% to 100% of mice died) following MES and INH-induced convulsions. Nonetheless, flower and stem extracts prevent the death of all mice following PLO-induced SE. All standard drugs protected 100% of mice from death. The severity of the seizures was inferred from the behavioral scale described in the materials and methods section. Mice were reached up to seizure stage 4. Stage 5 seizure was never attained in our PLO-induced groups within 30 min of observation, whereas 2–3 mice in the MES- and INH-inducer groups reached stage 5 seizure.

### 3.4 Effect of different doses of MP extract on the MDA values and SOD, CAT and GSH activity

**Fig. 3:**
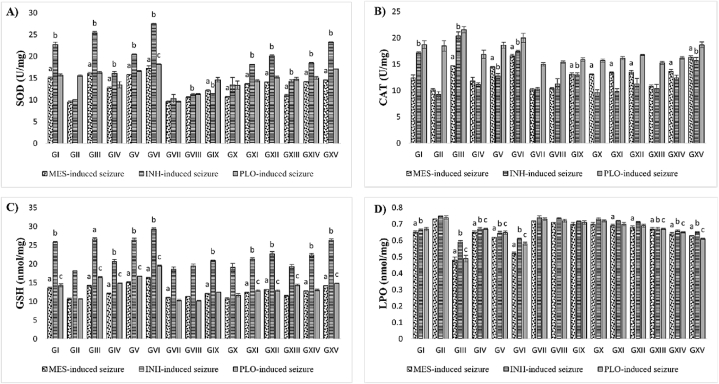
Effect of oral MP administration for 7 days on biochemical parameters of A) SOD, B) CAT, C) GSH, and D) LPO in seizure-induced mouse brain antioxidant status. Data are expressed as mean ± SEM (n = 5). Superscript alphabets represent the statistical significance analyzed by one-way ANOVA followed by Dunnett's test: ^a^p < 0.001 indicates a significant difference between the distil water + MES group and other groups in the MES-induced seizure model. ^b^p < 0.001 represents a significant difference between the distil water + INH group and other groups in the INH-induced seizure model. ^c^p < 0.001 denotes a significant difference between the distil water + INH group and other groups in the PLO-induced SE model.

The methanolic extract of MP100, 200, and 400 mg/kg doses showed significant (p < 0.001) decreases in MDA levels and increases in brain SOD, CAT, and GSH levels compared to seizure inducer groups (Fig. 3 A, B, and C). A comparison among the effects of different doses of MP extract, phenytoin, diazepam, and seizure inducer on the biochemical factors of brain tissue that are the common oxidative stress markers is shown in Fig. 3. As it is shown in Fig. 3 A and B, SOD and CAT levels have shown a significant increase (p < 0.001) compared to the MES and INH-induced seizures groups, but there is no significant change observed in MP-treated mice compared to PLO-induced seizure groups. SOD increased (0.06–21%) in leaf extracts, and (0.09–2.50%) in stem extracts, while CAT increased (1.39–33.9%) in leaf extract and (9.68–32%) in stem extracts, and (5.65–9%) in flower extracts, and 5.56% in root extracts. However, SOD and CAT levels increased in the order of leaves > stem > flower > root (Fig3 A and B), whereas CAT levels did not show any significant change in seizure inducer groups compared to the control groups. On the other hand, GSH levels increased significantly (p < 0.001) in MP-treated mice, especially leaves (2.22–37%) and stems (0.63–5%) extract potent GSH levels compared to all three seizures group (Fig. 3C). Fig. 3D, it is shown that the MDA level of the brain tissue has decreased significantly relative to the seizure inducer group (p < 0.001). Among all other parts of MP, leaves (2.71–20%) and stems (0.60–9%) have shown markedly decreased MDA levels in all three seizure inducers, while flower extract has exhibited significant lipid peroxidation activity at a dose of 200 and 400 mg/kg/BW in MES-induced seizure mice (Fig. 3D).

### 3.5 Effect of different doses of MP extract on the GABA level

**Fig. 4:**
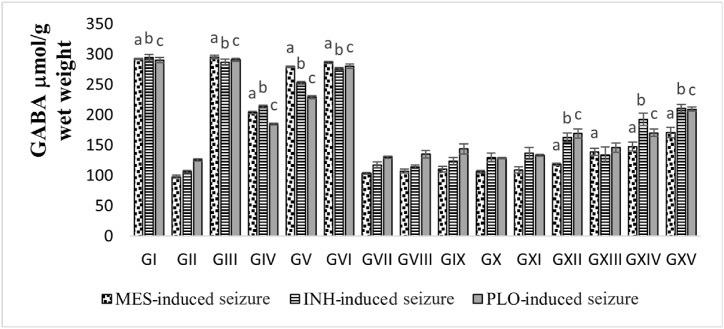
Effect of oral MP administration for 7 days on the GABA content of seizure mice brain antioxidant status. Data are expressed as mean ± SEM (n = 5). Superscript alphabets represent the statistical significance analyzed by one-way ANOVA followed by Dunnett's test: ^a^p < 0.001 indicates a significant difference between the distilled water + MES group and other groups in the MES-induced seizure model. ^b^p < 0.001 represents a significant difference between the distilled water + INH group and other groups in the INH-induced seizure model. ^c^p < 0.001 denotes a significant difference between the distilled water + PLO group and other groups in the PLO-induced seizure model.

The extract showed a significant increase in the GABA content in the order of leaves > stem > flower > root (Fig. 4), in a dose-dependent manner in all tree seizure inducer groups.

### 3.6 Correlation of GABA content, antioxidant enzymes, and antioxidant compounds in plant parts

**Fig. 5:**
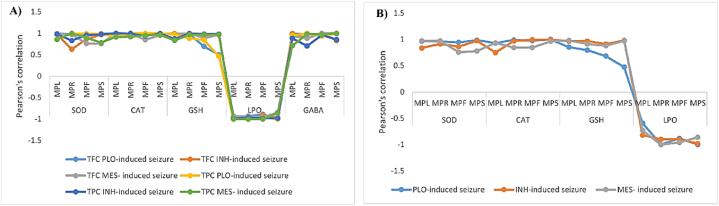
The correlation analysis of the phenolic compounds TFC and TPC with antioxidant profiles and GABA contents. A) Linear correlation coefficients observed between single phenolic compounds TFC and TPC and antioxidant capacities. B) The correlation analysis of GABA contents with the different antioxidant variables is shown here. SOD, CAT, and LPO were highly correlated with the GABA content in mice. MPL = leaf extracts; MPR = root extracts; MPF = flower extracts; MPS = stem extracts.

Pearson's correlation coefficient was evaluated to determine the correlation between all variables tested, TFC, TPC, and biological activities (SOD assay, CAT assay, GSH contents, lipid peroxidation (MDA level), and GABA content) (Fig. 5 A and B). Results showed significant positive correlations of GABA, CAT, SOD assay, and GSH content with the TPC and TFC of plant parts of MP extracts, but in the case of SOD and CAT, a non-significant (r = 0.627) correlation was observed with the TFC of root extracts. Correlation in the case of MDA levels, TPC, and TFC showed significant negative correlations for all parts of MP (Fig. 5). It was illustrated that higher levels of antioxidants in them would lower MDA levels and inhibit lipid peroxidation. The correlation of GABA and GSH content with TPC and TFC was moderately to highly significant in all parts of the plant. However, the correlation of GSH with TFC and TPC of stem extracts was found to be non-significant (r = 0.503 and r = 0.467, respectively) in PLO-induced mice, which could be explained by the fact that the presence of other non-flavanol compounds had a strong influence. Antioxidants like flavonoids and other non-flavanol compounds had a strong influence. The results indicate that antioxidant capacity and antioxidant enzymes are the major contributors to quenching radicals and oxidative stress and reducing seizure incidence, enhancing the importance of the antioxidant property of plant extracts. The Pearson correlation coefficient was positively high if significant = r ≥ 0.65. Accordingly, it has been reported that antioxidant compounds have anticonvulsant effects [36]. Furthermore, the effect of seizure inducers demonstrated in the biochemical assay supports the antioxidant activity of this antioxidant compound.

## 4 Discussion

Oxidative stress and the antioxidant system play an important role in the pathogenesis of epilepsy. This is because reactive oxygen species (ROS) from oxidative stress can cause neuronal injury and alter GABAergic neurotransmission [37,38], which is critical for the control of neuronal excitability and the prevention of seizures [[39], [40], [41]]. Studies have shown that oxidative stress can alter GABA synthesis and lead to the depletion of GABA content and other neurotransmitters, which may contribute to the development and progression of epilepsy [[42], [43], [44]]. Many studies have revealed that antioxidant compounds have properties that attenuate seizure events by restoring normal GABAergic signaling [[45], [46], [47], [48]]. However, the exact mechanisms linking oxidative stress and epilepsy still need to be elucidated. To evaluate the anticonvulsive and antioxidant properties of the MP extract, different flavonoids present in the plant were analyzed by chromatography and correlated with various antioxidant enzymes (SOD, CAT, and GSH), as well as TBARS (a lipid oxidation marker) and GABA content. This was performed to associate anticonvulsive activity with seizure-like behavior in three distinct models of epilepsy.

To our knowledge, this is the first study on the anticonvulsive activity of MP in a mouse model. Mice are commonly used in preclinical studies due to their physiological and genetic similarities to humans. Various seizure models, such as the pentylenetetrazol (PTZ) or maximal electroshock seizure (MES) models, are induced in mice to evaluate the effectiveness of potential anticonvulsant compounds [49,50]. Mice strains such as Swiss-albino mice have been widely used in seizures or epilepsy models for assessing anticonvulsant effects of plant extract [51,52]. This inbred mouse strain used in our seizure model is highly suitable due to its relatively high genetic homogeneity and moderate susceptibility to seizures induced by various methods. Their widespread use also allows for better comparison and interpretation of results across different studies. However, different mouse strains can increase seizure susceptibility due to genetic heterogeneity or treatment conditions [53,54]. By considering the susceptibility of different strains, it is important to explore whether certain strains exhibit stronger or weaker responses to the plant extracts, which could provide further insights into their effectiveness or potential limitations.

In the current study, MP extracts, including leaf and stem extracts, were able to reduce the onset of THLE and SE (stages 1–5), indicating that the extract possesses potent anticonvulsant activity against generalized tonic-clonic seizures (grand mal). Status epilepticus by PLO or INH-induced convulsion is associated with the inhibition of GABA synthesis [[55], [56], [57]]. Research has indicated that animals that have been given high doses of PLO or INH may experience continuous seizures, which have been linked to decreased levels of GABA in the brain [58,59]. Since INH is a GABAA receptor antagonist, it is possible that the extract may increase GABA concentration in the brain, and the GABA/benzodiazepine receptor complex mediates the anticonvulsant action of the MP extract. Furthermore, studies have shown that SE induced by pilocarpine alters brain antioxidant defenses by decreasing antioxidant enzymes [60,61]. Since the SE appears to be linked to low GABA levels and antioxidant enzymes in the brain, we therefore investigated the effect of the MP on GABA levels and antioxidant enzymes in the brain.

Antioxidant enzymes like CAT, SOD, and GSH play the most important role in scavenging oxidative stress-induced free radicals [62]. CAT is a mitochondrial and peroxisomal enzyme that breaks down H_2_O_2_ into water and oxygen [63]. CAT and hydro-peroxidase enzymes convert H_2_O_2_ and H_2_O_2_ to non-radical forms and function as natural antioxidants in the human body [64]. SOD enzyme plays a key role in detoxifying superoxide anions by catalyzing the conversion of O_2_∙− to H_2_O_2_ [18], which prevents oxidative stress-induced cellular damage [65]. Seizures are often preceded by abnormally low SOD and CAT levels [66]. In the current study, we observed that administration of MP leaf extracts increased SOD and CAT content in a dose-dependent manner, as reported during supplementation with MP in MES, INH, and PLO-induced seizures in mice. SOD and CAT activities can protect against seizures induced by INH and MES. However, there were no changes in SOD and CAT levels during the acute phase of PLO- induced seizures, suggesting that these enzymes cannot be activated during their phase of seizures and, therefore, other antioxidant systems can be responsible for the inhibition of PLO-induced acute convulsive activity.

GSH is known as the most prevalent and important intracellular non-protein thiol [67]. It is an endogenous antioxidant that plays a vital role as a free-radical scavenger that protects cells against oxidative damage [68]. Modification of intracellular levels of GSH has also been shown to regulate seizure susceptibility and neuronal survival [69]. In the present study, GSH content decreased in seizer-induced control groups of mice, indicating that there was an increased generation of free radicals, and that is why the reduced glutathione was depleted during the process of combating oxidative stress. In addition, at a dose of 200 and 400 mg/kg, the leaves and stems extract showed significant increases in brain GSH levels in both INH- and MES-induced seizures mice compared to respective controls.

Previous studies demonstrated that peroxidation of membrane polyunsaturated fatty acids produces toxic MDA, which compromises membrane lipids, and matrix dynamics and results in the progression of seizures in PLO-induced mice [66]. It has been reported that PLO-induced mice showed a significant imbalance between antioxidants and free radicals, as evidenced by elevated MDA levels and decreased GSH levels [62]. The high concentration of hydro-peroxides in tissues suggests that the hippocampal cells are more vulnerable to damage during the acute period of seizures [64]. The brain is particularly susceptible to peroxidation due to the simultaneous presence of high levels of polyunsaturated fatty acids and iron [70], which is the target of free radical damage. In the current study, we found a rise in the lipid peroxidation level in the brain homogenate of mice with seizures. This is reflected by a rise in TBARS levels, which may be related to the intermediate free radicals formed during seizures induced by PLO.

MP leaf extract has been found to have protective effects against seizures, which could be explained by an elevated endogenous GABA concentration. Previous studies have shown that drugs that increase the brain contents of GABA have exhibited anticonvulsant activity against MES and pentylenetetrazol seizures [71]. This suggests that the higher anticonvulsant activity of MP leaf extract is due to the elevation of brain GABA content. MP significantly inhibited generalized tonic-clonic seizures in MES, PLO, and INH tests, suggesting the presence of anticonvulsant compounds. Polyphenols, or flavonoids, are compounds that exhibit potent antioxidant properties [72,73]. In the current study, antioxidant compounds from various parts of MP showed a significant positive correlation with GABA content. It has been reported that free radical generation induces seizure activity by inactivating glutamine synthesis or inhibiting glutamate decarboxylase, leading to a decrease in GABA turnover and, in turn, facilitating abnormal excitation [74]. GABA content is significantly more correlated to total flavonoids than the total phenolic content of all parts of MP, which may be due to the presence of flavonoids in the MP plant playing an important role in increasing GABA content and reducing seizure incidents. It is notable from the results that in MES and INH, none of the deaths were observed, while in PLO, higher doses of MP protected more than 70% of animals from convulsive mortality.

Our experimental results indicate the presence of an especially high concentration of an anticonvulsant compound in the MP leaf extracts. This compound, including QU and ECH, was found in the highest amounts, while VA and FA were found in the lowest amounts. *Studies have suggested that flavonoids inhibit voltage-gated Na + channels, activate by Ca2+ and K+ channels*, stimulate GABAergic inhibition, and exhibit antioxidant actions via modulation of nitric oxide and xanthine oxidase pathways, and one or more of these mechanisms are involved in the suppression of epileptic seizures [75,76]. Flavonoids like apigenin, QU, KF, rutin, naringenin, hesperidin, ECH, and their derivatives have been found to have protective roles in epileptic disorders [66,77]. In our studies, the initiation time and duration of convulsions were highest with the leaf extract.

In our study, we found MP leaves contained polyphenolics and flavonoids like AP, VN, KF, and QU MP. These polyphenols and flavonoids may directly inhibit convulsions, block glutamatergic transmission, and reduce the onset latency of seizures and central nervous system excitotoxicity [78]. KF is also reported to reverse the oxidative stress-induced-decrease of hippocampal BDNF and cAMP Response Element Binding Protein expression, leading to hippocampal neurogenesis and ameliorating depression and cognitive deficits [79,80]. QU is an antioxidant that inhibits ROS production and acts as an NMDA receptor antagonist [81]. FA is a phenolic phytochemical with antioxidant [82,83], and neuroprotective properties found in the MP leaf extract. VA has been shown to reduce ROS induction and lipid peroxidation in epileptic mice [84,85], suggesting that antioxidant activity might play some role in its beneficial effects. MP extracts contain antioxidant properties, that may reduce the level of oxidative stress in the body. By reducing oxidative stress, plant extracts can potentially modify the course of epileptogenesis, which refers to the development and progression of epilepsy, as well as reduce the occurrence of seizures and the severity of their symptoms, as observed with MES, INH, and PLO-induced SE.

Recently, it has been reported that some flavonoids can cross the blood-brain barrier and bind to benzodiazepine sites, producing anticonvulsant and central depressant effects [86,87]. Antioxidants have neuroprotective effects, showing increased levels of endogenous antioxidant enzyme activities, increased non-enzymatic antioxidant levels, and decreased markers of oxidative stress damage [88]. As per our results, doses of MP leaf extract 100, 200, and 400 are likely to abolish convulsions due to the binding of some bioactive flavonoids to benzodiazepine sites or elevating GABA concentrations, thereby inhibiting hyperexcitation of neuronal discharges.

## 5 Conclusion

Overall, MP leaf and stem extracts have an anticonvulsive effect on MES, INH, and PLO-induced seizure mice, suggesting an antioxidant mechanism. Moreover, there is a positive correlation observed with the involvement of GABA content with antioxidant mechanisms and an anticonvulsive effect, which suggests that MP extracts exert effects through modulating the GABAergic system in the mouse brain, which can be predicted due to the presence of different categories of phytoconstituents (phenolics, terpenoids, and steroids). Therefore, the leaves and stems of MP could be a potential source in the management of convulsions. Further research is required to determine whether MP-derived antioxidants that attenuate oxidative stress have an impact on neuroprotection.

## Author contribution statement

S. M. kamruzzaman: Performed the experiments; Wrote the paper.

Latifa Bulbul: Conceived and designed the experiments; Analyzed and interpreted the data; Wrote the paper.

Md Zahir Alam: Performed the experiments.

Md Mostafizur Rahman: Analyzed and interpreted the data; Contributed reagents, materials, analysis tools or data.

**Funding statement**

This research did not receive any specific grants from public, commercial, or non-profit funding agencies.

## Data availability statement

Data will be made available on request.

## Declaration of competing interest

The authors declare that they have no known competing financial interests or personal relationships that could have appeared to influence the work reported in this paper.
